# Low protein intake, physical activity, and physical function in European and North American community-dwelling older adults: a pooled analysis of four longitudinal aging cohorts

**DOI:** 10.1093/ajcn/nqab051

**Published:** 2021-04-07

**Authors:** Nuno Mendonça, Linda M Hengeveld, Marjolein Visser, Nancy Presse, Helena Canhão, Eleanor M Simonsick, Stephen B Kritchevsky, Anne B Newman, Pierrette Gaudreau, Carol Jagger

**Affiliations:** EpiDoC Unit, CEDOC, NOVA Medical School, Universidade Nova de Lisboa (UNL), Lisbon, Portugal; Comprehensive Health Research Centre (CHRC), NOVA Medical School, Lisbon, Portugal; Population Health Sciences Institute, Newcastle University, Newcastle-upon-Tyne, United Kingdom; Department of Health Sciences, Faculty of Science, Amsterdam Public Health Research Institute, Vrije Universiteit Amsterdam, Amsterdam, The Netherlands; Department of Health Sciences, Faculty of Science, Amsterdam Public Health Research Institute, Vrije Universiteit Amsterdam, Amsterdam, The Netherlands; Research Centre on Aging, CIUSSS de l'Estrie-CHUS, Sherbrooke, Quebec, Canada; Faculty of Medicine and Health Sciences, University of Sherbrooke, Sherbrooke, Quebec, Canada; Centre de recherche de l'Institut universitaire de gériatrie de Montréal, Montréal, Quebec, Canada; EpiDoC Unit, CEDOC, NOVA Medical School, Universidade Nova de Lisboa (UNL), Lisbon, Portugal; Comprehensive Health Research Centre (CHRC), NOVA Medical School, Lisbon, Portugal; National Institute on Aging Intramural Research Program, Baltimore, MD, USA; Sticht Center on Aging, Wake Forest School of Medicine, Winston-Salem, NC, USA; Center for Aging and Population Health, Department of Epidemiology, University of Pittsburgh, PA, USA; Department of Medicine, University of Montréal, H3T 1J4, Montréal, Quebec, Canada; Research Centre of the Centre hospitalier de l'Université de Montréal (CHUM), H2X 0A9, Montréal, Quebec, Canada; Population Health Sciences Institute, Newcastle University, Newcastle-upon-Tyne, United Kingdom

**Keywords:** protein, walking speed, gait speed, physical activity, joint models, PROMISS, older adults, one-stage meta-analysis

## Abstract

**Background:**

Dietary protein may slow the decline in muscle mass and function with aging, making it a sensible candidate to prevent or modulate disability progression. At present, studies providing reliable estimates of the association between protein intake and physical function, and its interaction with physical activity (PA), in community-dwelling older adults are lacking.

**Objectives:**

We investigated the longitudinal relation between protein intake and physical function, and the interaction with PA.

**Methods:**

We undertook a pooled analysis of individual participant data from cohorts in the PROMISS (PRevention Of Malnutrition In Senior Subjects in the European Union) consortium (the Health Aging and Body Composition Study, Quebec Longitudinal Study on Nutrition and Successful Aging, Longitudinal Aging Study Amsterdam, and Newcastle 85+) in which 5725 community-dwelling older adults were followed up to 8.5 y. The relation between protein intake and walking speed was determined using joint models (linear mixed-effects and Cox proportional hazards models) and the relation with mobility limitation was investigated using multistate models.

**Results:**

Higher protein intake was modestly protective of decline in walking speed in a dose-dependent manner [e.g., protein intake ≥1.2 compared with 0.8 g/kg adjusted body weight (aBW)/d: β = 0.024, 95% CI: 0.009, 0.032 SD/y], with no clear indication of interaction with PA. Participants with protein intake ≥0.8 g/kg aBW/d had also a lower likelihood of incident mobility limitation, which was observed for each level of PA. This association seemed to be dose-dependent for difficulty walking but not for difficulty climbing stairs. No associations between protein intake and other mobility limitations transitions were observed.

**Conclusions:**

Higher daily protein intake can reduce physical function decline not only in older adults with protein intake below the current RDA of 0.8 g/kg BW/d, but also in those with a protein intake that is already considered sufficient. This dose-dependent association was observed for each level of PA, suggesting no clear synergistic association between protein intake and PA in relation to physical function.

## Introduction

Life expectancy has reached 81.0, 82.1, and 78.7 y in the European Union, Canada, and United States, respectively (1–3). However, the increase in healthy life years (HLYs)—the number of years an individual can expect to live disability-free—has not kept pace (4). Bridging the gap between life expectancy and HLYs by compressing morbidity into the later years of life is of special interest, not only to increase quality of life, but also to relieve the immense strain on the health care systems of developed countries.

Diet is a major modifiable risk factor for the development and management of a range of age-related diseases that comprise the leading causes of morbidity, disability, and death (5, 6). Specifically, dietary protein can slow the decline of muscle mass and function with aging, making it a sensible candidate to prevent or modulate disability progression (7–9). For example, community-dwelling older adults (70–79 y) with lower protein intake [<1 g/kg body weight (BW)/d] from the Health Aging and Body Composition Study (Health ABC) study had an increased risk of mobility limitation over 6 y compared with those with higher protein intake (≥1 g/kg BW/d) (10). In the Newcastle 85+ Study, community-dwelling very old adults (≥85 y) with protein intake <1 compared with ≥1 g/kg adjusted body weight (aBW)/d had lower muscle strength over 5 y (9). However, at present, evidence for an association between protein intake and (performance-based) loss of physical function in older adults is limited. One study in older females showed that higher protein intake was associated with a slower rate of physical function decline (11), but other studies did not find protein intake to be associated with less decline in physical function (9, 12–15).

Inadequate protein intake is a cause for concern in older adults because protein intake is lower in older males (87 g/d) and females (69 g/d) than in their younger counterparts (97 g/d and 73 g/d, respectively) (16, 17) due to multimorbidity, tooth loss, changes in deglutition, appetite loss, and loss of functional independence (18). Additionally, a higher prevalence of disease-related tissue catabolism and inflammation, and anabolic resistance can offset protein requirements (19, 20). Expert groups, such as PROT-AGE, have suggested a possible synergistic protective effect of protein and physical activity (PA) on age-related loss of muscle strength and muscle function (18). This would be anticipated if higher PA and higher protein intake together were necessary to optimally stimulate the rate of muscle synthesis, which might consequently decrease the rate of loss of muscle mass and muscle function and slow the rate of functional decline in older adults. However, only a few cohort studies have addressed this issue (9, 12) and evidence from randomized controlled trials (RCTs) is inconclusive (21–23). This could be due to a lack of statistical power in individual studies, which can be overcome by pooling individual participant data from multiple studies. We hypothesized that higher protein intake (especially ≥1 g/kg aBW/d), alone or in combination with PA, is protective against physical function loss. We therefore aimed to: *1*) investigate the prospective relation between protein intake and physical function (using objective and subjective measures); and *2*) explore the interaction between protein intake and PA in relation to physical function in 4 longitudinal aging cohorts in the PROMISS (PRevention Of Malnutrition In Senior Subjects in the European Union) consortium.

## Methods

### Cohorts included

**FIGURE 1:**
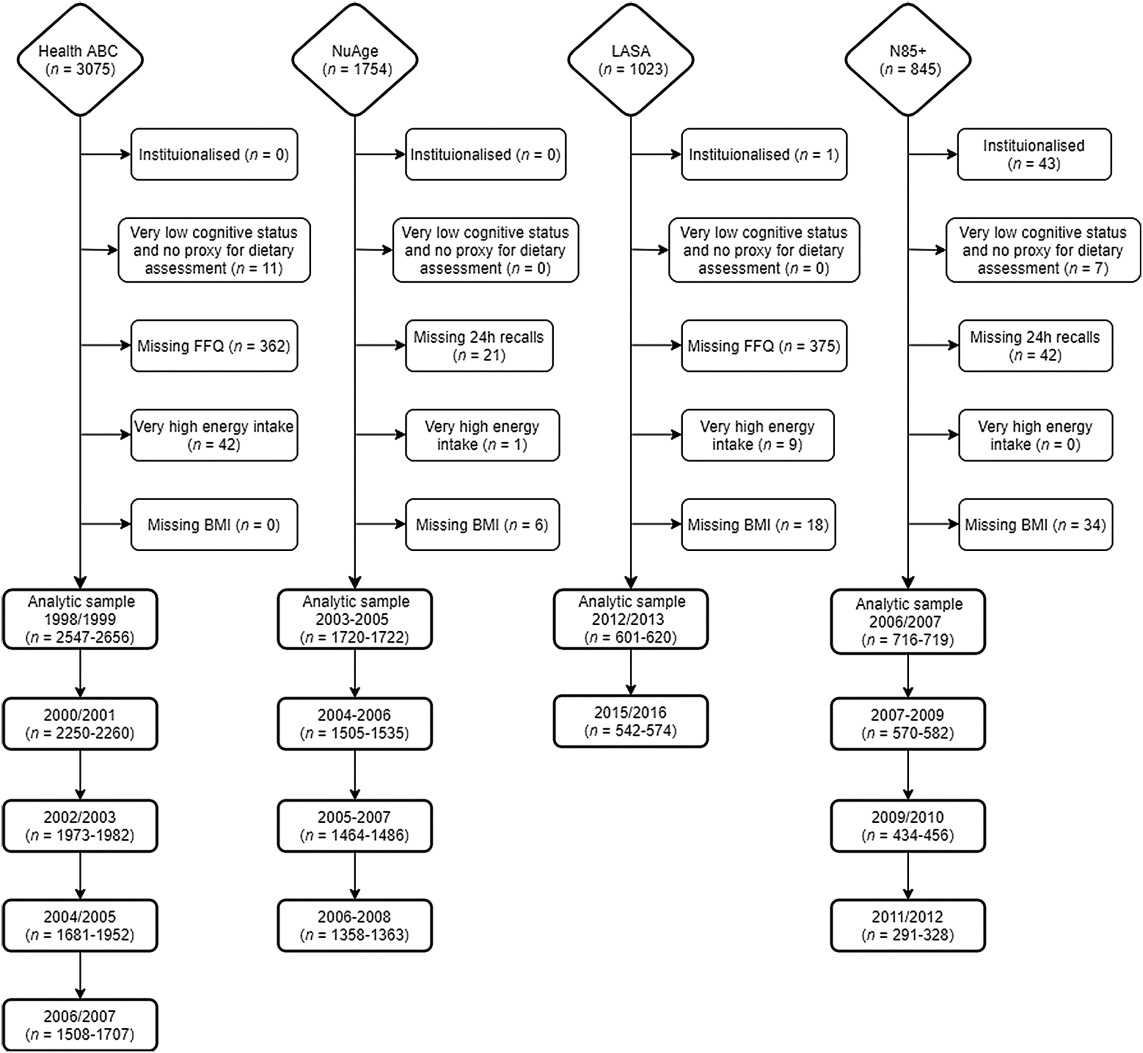
Flowchart of the 4 longitudinal aging cohorts included and the exclusion criteria for the analytic sample. Very low cognitive status was defined by a Mini-Mental State Examination score <18 or having dementia. Very high energy intakes were defined as >3500 kcal/d for females or >4000 kcal/d for males. The final sample depends on the availability of walking speed, self-reported ability to walk >200 m, or ability to climb stairs (hence a range of *n* is given). Health ABC, Health, Aging and Body Composition Study; LASA, Longitudinal Aging Study Amsterdam; N85+, Newcastle 85+ Study; NuAge, Quebec Longitudinal Study on Nutrition and Successful Aging.

As part of the PROMISS consortium, 4 longitudinal prospective observational studies were included: *1*) the Health, Aging and Body Composition Study (Health ABC); *2*) the Quebec Longitudinal Study on Nutrition and Successful Aging (NuAge); *3*) the Longitudinal Aging Study Amsterdam (LASA); and *4*) the Newcastle 85+ Study (**Figure 1**). These studies are described in detail elsewhere (24–27). Briefly, Health ABC is a longitudinal cohort study that included 3075 well-functioning community-dwelling black and white males and females aged 70–79 y living in the United States at baseline. Participants were recruited from Medicare-eligible residents in the metropolitan areas of Memphis, Tennessee, and Pittsburgh, Pennsylvania, between April 1997 and June 1998 and followed annually (clinic visit) or every 6 mo (telephone interview) for 16 y (24). We used year 2 (baseline) and follow-up data until year 10. NuAge is a longitudinal cohort that recruited 1793 generally healthy community-dwelling males and females aged 67–84 y living in Montreal and Sherbrooke areas (Quebec, Canada) in 2003–2005 and followed them annually (clinic visit) or every 6 mo (telephone interview) for 3 y (27). We used data from baseline and all 3 follow-up waves for all participants who agreed to be part of the NuAge Database and Biobank (*n* = 1754 at baseline). LASA is an ongoing nationally representative longitudinal study of older males and females aged ≥55 y residing in The Netherlands. The study started in 1992/93 (*n* = 3107) and participants were followed every 3 y until 2018/2019 (most recent wave: wave J). Two additional cohorts were recruited from the same sampling frames at 10 y (2002/2003, *n* = 1002) and 20 y (2012/2013, *n* = 1023) after the baseline (25). We used data from wave 3B (2012/2013), the Nutrition and Food-related Behaviour substudy (2014/2015) and wave I (2015/2016). The Newcastle 85+ Study is a longitudinal population-based study that approached all people turning 85 y in 2006/2007 (born in 1921) in Newcastle and North Tyneside, United Kingdom. At baseline, there were 845 very old males and females who agreed to a health assessment and a review of their GP records (26), and who were re-examined after 18, 36, and 60 mo. We used data from baseline and all 3 follow-up waves.

### Exclusion criteria

We excluded participants (Figure 1) who were institutionalized (*n* = 44), had very poor cognitive status (score <18 in the Mini-Mental State Examination or with diagnosed dementia) and no proxy for dietary assessment (*n* = 18), had missing dietary intake data (*n* = 800), had very high reported energy intake, that is, >3500 kcal/d for females or >4000 kcal/d for males (*n* = 52), or had no data on BMI (*n* = 58). The analytic sample comprised 5658–5725 (depending on the outcome, i.e., walking speed, difficulty walking >200 m, or difficulty climbing stairs) community-dwelling participants aged ≥55 y.

### Missing and nonmissing data on walking speed and mobility limitation

Older adults who were smokers, had lower energy intake, low PA, and had more mobility limitation had more missing data on walking speed. Those with low education, poor cognitive status, non–alcohol drinkers, low PA, and slower walking speed had more missing data on mobility limitation (**Supplemental Table 1**).

### Dietary assessment

Dietary intake was assessed by an FFQ in Health ABC and LASA, and by multiple 24-h recalls in NuAge and Newcastle 85+. In Health ABC, a 108-item interviewer-administered FFQ (28) that reflected the preceding 12 mo was used at the first follow-up (year 2; 1998/1999) and served as the baseline for the present study. In LASA, a validated (29) self-administered 238-item FFQ (30) that reflected the preceding 4 wk was used. In LASA, dietary intake was not assessed at a regular measurement cycle, but in the Nutrition and Food-related Behavior Study 2014–2015 substudy. In NuAge, 3 recalls per wave were collected by trained registered dietitians on 2 randomly chosen weekdays and 1 weekend day, 1 face-to face and 2 by telephone (27). Portion sizes were estimated with the aid of portion size models. In the Newcastle 85+ Study, two 24-h recalls were collected by trained research nurses on 2 nonconsecutive weekdays ≥1 wk apart (31, 32) and portion sizes were estimated with the aid of a photographic food atlas. In all studies, energy and protein intake were calculated by using country-specific food composition databases. In studies that assessed dietary intake using 24-h recall, individual intakes of protein and energy were averaged within the 2 (Newcastle 85+) or 1 or 3 (NuAge) measurements. Dietary intake was available at baseline for Health ABC, LASA, and Newcastle 85+, and at baseline and waves 2, 3, and 4 for NuAge.

For participants with an undesirable body weight (BW), BW was adjusted to be within the desired BMI for older adults of 22–27 kg/m^2^ and calculated as previously described (33, 34). By this, we attempted to control for the deficit and excess in BW in underweight and overweight people, respectively. All variables are described in **Supplemental Table 2**. Protein intake was expressed as a categorical variable using cut points of <0.8, 0.8–0.99, 1.0–1.19, and ≥1.2 g/kg BW/d and g/kg aBW/d. These cutoffs were based on expert recommendations for optimal protein intake (18, 20), on currently used RDAs for protein [e.g., 0.8 is recommended by the European Food Safety Authority (EFSA) (35) and the Institute of Medicine (IoM) (36), 1.0 by the European DACH (Germany, Austria, and Switzerland) countries (37), and 1.2 by the European Nordic countries (38)], and on previously published studies on protein intake in older adults (9, 24, 34, 39–43).

### Physical activity

In Health ABC, total PA was measured (at baseline) by a specifically designed self-reported questionnaire as described previously (44). Participants indicated whether they had performed exercise (part of daily routine or leisure-time) in the past 7 d and how long they spent in each activity. These included gardening, heavy chores, light housework, grocery shopping, laundry, climbing stairs, walking, and moderate- and high-intensity exercise. A metabolic equivalent (MET) value in kilocalories per week per kilogram BW was determined for each activity. A total PA score was calculated as the sum of the MET values for each activity multiplied by the participant's BW in kilograms. In NuAge, PA was measured using the self-reported Physical Activity Scale for the Elderly (45). This validated questionnaire included work-related, household, and leisure time activities during a 1-wk period. The total PA score is derived from multiplying the amount of time spent on each activity (in hours per week) by the item weights and summing over all activities (46). PA in LASA was measured with a validated interviewer-administered questionnaire that estimates the frequency, duration, and intensity of specific activities in the previous 2 wk (47). These included walking, cycling, light and heavy household work, and first and second sport. MET scores were assigned to each activity based on published lists of MET scores (48). For each activity, the frequency, duration in minutes, and MET score were multiplied and then divided by 14 d. The minutes spent per activity per day were summed to a total PA score (minutes/day × MET). In Newcastle 85+, a validated purposely designed physical activity questionnaire included questions on how frequently [≥3 times/wk (score of 3), 1–2 times/wk (score of 2), 1–3 times/mo (score of 1), and hardly ever (score of 0)] the participants engaged in mildly energetic (e.g., light gardening, light housework), moderately energetic (e.g., moderate gardening, walking at moderate pace, heavy housework), and highly energetic (e.g., heavy gardening, swimming, cycling) activities. The resulting total PA score was calculated as 3 × highly energetic activities + 2 × moderately energetic activities + mildly energetic activities (49). PA was transformed into cohort-specific tertiles (categorized as low, medium, and high) at baseline, which were used to categorize the PA score for subsequent waves.

### Walking speed

Walking speed was used as an objective (performance-based) measure of physical function. Walking speed (meters per second) was measured as the time (seconds) taken to walk a preset distance—20 m in Health ABC, 4 m in NuAge, and 3 m in LASA—at usual speed and calculated as distance (meters) divided by time (seconds). In Newcastle 85+, Timed-Up-and-Go (TUG) was measured instead of walking speed. To harmonize Newcastle 85+ with the other cohorts, the formula [6/TUG (s)]×1.62 was used to yield walking speed (m/s), as recommended (50).

### Mobility limitation

Self-reported difficulty walking >200 m and climbing stairs were used as subjective measures of physical function and are here defined under the broad heading of mobility limitations. In Health ABC, difficulty walking was determined by asking the participants if they had any difficulty walking a quarter mile (400 m) because of a health or physical problem; difficulty in climbing stairs was determined by asking the participants if they were able to climb 1 flight of stairs (∼10 steps). In NuAge, participants were asked if their health status prevented them from walking >200 m and if they could go up and down a staircase (∼10 steps). In LASA, participants were asked if they had any difficulty walking for 5 min outside the house without stopping (equivalent to 240 m if walking speed was 0.8 m/s), and if they could walk up and down a staircase of 15 steps without resting. In Newcastle 85+, participants were asked if they had any difficulty walking ≥400 yards (366 m) and if they could go up and down stairs. Difficulty walking was harmonized by creating a binary variable: able to walk >200 m without difficulty, or not (with some difficulty, with help, or unable). Difficulty climbing stairs was harmonized as being able to climb stairs without difficulty or not (with some difficulty, with help, or unable).

### Mortality

In Health ABC, deaths were confirmed by death certificate and review of hospital records, obituaries, and interviews with family members. Time to death was calculated as the time between age at baseline (1998/1999) and age of death or September 30, 2014, whichever came first. In NuAge, deaths were recorded during data collection. Age at baseline (2003–2005) and deaths (censored at May 3, 2010) were used to calculate the time to death. LASA was linked with mortality data of Statistics Netherlands (Centraal Bureau voor de Statistiek). Time to death was calculated as the time from baseline age (2012/2013) to death or July 22, 2018, whichever came first. In Newcastle 85+, information on date of death was provided by National Health Service Digital UK, and time to death was calculated as the time between baseline age (2006–2007) and age at death (censored at January 16, 2018).

The methods used to assess education, BMI, smoking, alcohol drinking, multimorbidity, and cognitive status in each cohort are described in the **Supplemental Methods**. All variables used and their operationalization are described in Supplemental Table 2.

### Ethics approval and consent to participate

Health ABC was approved by the institutional review boards of the University of Tennessee, Memphis, Tennessee, and the University of Pittsburgh, Pittsburgh, Pennsylvania. LASA was approved by the medical ethics committee of the VU University Medical Center. The Newcastle 85+ Study was approved by the Newcastle & North Tyneside Local Research Ethics Committee 1. Signed consent was obtained from each participant, and a signed consultee approval was obtained whenever the patient lacked capacity. NuAge was approved by the ethics committees of both the University Institutes of Geriatrics of Sherbrooke and Montréal and the Research Ethics Board of the McGill University Health Centre. All studies were conducted in line with the Declaration of Helsinki, and all participants provided written informed consent and/or a signed consultee approval was obtained whenever the participant lacked capacity.

### Statistical analyses

Data cleaning, quality control, and harmonizing were performed separately for each cohort prior to merging. Because the methods to assess energy intake and walking speed were different between cohorts, these were transformed into cohort-specific *z*-scores [*z* = (x − µ)/σ)]. For walking speed, the baseline mean and SD was used to create *z*-scores for the other waves. Normality was assessed by Q-Q plots: normally distributed variables are presented as means and SDs, non-Gaussian distributed variables as medians and IQRs, and categorical data as percentages (with corresponding frequency). To determine the relation between protein intake and walking speed we performed hierarchical linear mixed effects models with the lme4 package (version 1.1-20) (51) and Cox proportional hazards for time-to-event data (mortality) with the survival package (version 2.43-3) (52). Data missing at random were accounted for in the linear mixed models. However, as in any longitudinal cohort of aging, attrition was high (53) and failure to account for mortality (nonrandom attrition) would likely result in biased estimates toward the null (54). These outcomes (walking speed and mortality) are typically analyzed separately, but joint models analyze the 2 outcomes together with shared parameters in a single likelihood function (maximum likelihood estimation). We therefore fitted joint models with the JoineRmeta package (version 0.1.2) (55) in R v3.6.3. To determine the association between protein intake and transitions in mobility limitation (i.e., difficulty walking >200 m and difficulty climbing stairs) we fitted multistate models with 3 states: no limitation, limitation, and death (absorbing state); the illness-death model with the allowed transitions is shown in **Supplemental Figure 1**. Multistate models describe the movement of an individual between a number of finite states in a continuous time stochastic process under the Markov assumption that the next state is only influenced by the current state (56, 57). Multistate models were fitted with the msm package in R v3.2.2 (58). For walking speed and mobility limitation (i.e., difficulty walking and difficulty climbing stairs), we fitted 3 models with increasing complexity with protein intake <0.8, 0.8–0.99, 1.0–1.19, and ≥1.2 g/kg BW/d (actual BW) and g/kg aBW/d (adjusted BW) as the primary exposure. Separate models for walking speed and mobility limitation were also stratified by PA level. Model l included protein intake, study (random effect in the joint models), age, sex, and education; Model 2 was adjusted for the previous variables plus energy intake, smoking, and alcohol drinking; Model 3 was further adjusted for multimorbidity, cognitive status, and PA (except if the model was stratified by PA level). Point estimates and CIs were used to assess statistical and clinical significance. Results are presented as βs and 95% CIs for the joint models, and as HRs and 95% CIs for the multistate models.

## Results

### Health and sociodemographic characteristics and functional status

The analytic sample consisted of 5725 participants with a median age of 75.0 y (IQR: 71.6–79.0 y) at baseline, 53% of which (*n* = 3035) were females (**Table 1**). Most participants were from the Health ABC study (46.5%), followed by NuAge (30.1%), Newcastle 85+ (12.6%), and LASA (10.8%) at baseline (Table 1, Figure 1). At baseline, 28%, 23%, 21%, and 28% of the participants had protein intake <0.8, 0.8–0.99, 1.0–1.19, and ≥1.2 g/kg aBW/d, respectively (Table 1). The protein intake groups were of similar age (age was “statistically” different but this difference was not meaningful), sex ratio, education, multimorbidity, cognition, and smoking status, but differed in alcohol consumption and energy intake (higher protein intake category included a higher proportion of alcohol drinkers and higher energy intake) and in PA (e.g., 29.6% of those with protein intake ≥1.2 g/kg aBW/d had a low level of PA, whereas this was 36.7% in those with protein intake <0.8 g/kg aBW/d). Participants with higher protein intake had slightly faster walking speed (*z*-score) and were less likely to have mobility limitation (Table 1). Maximum follow-up time was 8.5 y (mean: 2.5 ± 2.4 y). Walking speed decreased modestly with age (from 1.06 ± 0.28 m/s at baseline to 1.01 ± 0.25 m/s at wave 4, and 1.03 ± 0.23 m/s at wave 5), and the proportion of participants with mobility limitation increased with age [e.g., 18% (*n* = 1031) of participants had difficulty walking >200 m at baseline, and this increased to 30.2% (*n* = 1092) at wave 4 and 35.4% (*n* = 605) at wave 5] (**Supplemental Figure 2**, **Table 2**). Health and sociodemographic characteristics by protein intake categories and cohort or PA are shown in **Supplemental Tables 3** and **4**.

**TABLE 1:** Baseline health and sociodemographic characteristics of participants by protein intake category^1^

			Protein intake, g/kg aBW/d				
	All (*n* = 5725)	Missing, %	<0.8 (*n* = 1579)	0.8–0.99 (*n* = 1335)	1.0–1.19 (*n* = 1218)	≥1.2 (*n* = 1593)	*P*
Age, y, median (IQR)	75.0 (71.6–79.0)	0.0	75.0 (72.0–79.0)	75.0 (72.0–79.2)	75.0 (71.0–79.0)	74.0 (70.8–79.0)	<0.001
Females, % (*n*)	53.0 (3035)	0.0	53.8 (849)	53.7 (717)	53.5 (652)	51.3 (817)	0.448
Cohort, % (*n*)		0.0					<0.001
Health ABC	46.5 (2660)		66.1 (1044)	44.8 (598)	37.6 (458)	35.2 (560)	
NuAge	30.1 (1726)		16.2 (256)	32.5 (434)	36.9 (450)	36.8 (586)	
LASA	10.8 (620)		5.0 (79)	8.1 (108)	13.7 (167)	16.7 (266)	
N85+	12.6 (719)		12.7 (200)	14.6 (195)	11.7 (143)	11.4 (181)	
Education, % (*n*)		0.1					0.071
Low	31.9 (1822)		31.1 (490)	33.2 (443)	30.1 (366)	32.8 (523)	
Medium	37.7 (2156)		36.8 (580)	36.4 (486)	37.8 (460)	39.5 (630)	
High	30.4 (1741)		32.1 (505)	30.4 (406)	32.1 (390)	27.6 (440)	
Multimorbidity, % (*n*)	51.0 (2784)	4.6	49.6 (763)	52.6 (663)	53.0 (605)	49.6 (753)	0.144
Cognition, % (*n*)		2.9					0.025
Low	29.9 (1660)		31.3 (472)	29.8 (388)	29.9 (357)	28.5 (443)	
Medium	41.7 (2319)		39.4 (594)	40.9 (532)	40.6 (484)	45.5 (709)	
High	28.4 (1579)		29.4 (443)	29.2 (380)	29.4 (351)	26.0 (405)	
Smokers, % (*n*)	8.5 (488)	0.3	8.1 (127)	8.4 (112)	8.6 (105)	9.1 (144)	0.784
Alcohol drinkers, % (*n*)	44.5 (2545)	0.0	37.7 (596)	42.6 (569)	48.4 (589)	49.7 (791)	<0.001
Energy intake, *z*-score (mean ± SD)	−0.0 ± 1.0	0.0	−0.8 ± 0.7	−0.2 ± 0.7	0.2 ± 0.8	0.8 ± 0.9	<0.001
Physical activity, % (*n*)		0.1					<0.001
Low	32.3 (1849)		36.7 (580)	30.0 (400)	32.6 (397)	29.6 (472)	
Medium	33.8 (1935)		34.1 (539)	35.5 (473)	34.7 (422)	31.5 (501)	
High	33.9 (1937)		29.1 (460)	34.5 (460)	32.7 (398)	38.9 (619)	
Walking speed, *z*-score (mean ± SD)	−0.0 ± 1.0	0.9	−0.1 ± 1.0	0.0 ± 1.0	0.0 ± 1.0	0.1 ± 1.0	0.005
Mobility limitations, % (*n*)							
Difficulty walking >200 m	18.0 (1031)	0.0	22.2 (351)	17.8 (238)	16.3 (198)	15.3 (244)	<0.001
Difficulty climbing stairs	21.3 (1208)	0.9	25.0 (390)	21.6 (286)	19.1 (231)	19.1 (301)	<0.001

1 Cognition was assessed with the Mini-Mental State Examination. Smokers and alcohol drinkers represent current consumers. *z*-scores and tertiles are cohort-specific. Nondifference between protein intake categories was assessed with χ^2^ test for categorical variables and ANOVA/Kruskal–Wallis for continuous variables along with the effect size and SD or 95% CI. aBW, adjusted body weight; Health ABC, Health, Aging and Body Composition Study; LASA, Longitudinal Aging Study Amsterdam; N85+, Newcastle 85 + Study; NuAge, Quebec Longitudinal Study on Nutrition and Successful Aging.

**TABLE 2:** Sociodemographic characteristics and functional outcomes by wave of follow-up^1^

	Baseline (*n* = 5725)	Wave 2 (*n* = 5337)	Wave 3 (*n* = 4419)	Wave 4 (*n* = 3980)	Wave 5 (*n* = 1865)
Age, y, median (IQR)	75.0 (71.6–79.0)	76.0 (73.0–80.0)	78.4 (76.0–82.0)	80.0 (77.2–83.3)	82.0 (80.0–84.0)
Females, % (*n*)	53.0 (3035)	53.5 (2856)	54.3 (2398)	54.6 (2172)	54.9 (1023)
Cohort, % (*n*)					
Health ABC	46.5 (2660)	46.6 (2487)	53.2 (2353)	52.4 (2085)	100.0 (1865)
NuAge	30.1 (1726)	31.2 (1666)	36.4 (1610)	39.4 (1567)	0.0 (0)
LASA	10.8 (620)	11.3 (602)	0.0 (0)	0.0 (0)	0.0 (0)
N85+	12.6 (719)	10.9 (582)	10.3 (456)	8.2 (328)	0.0 (0)
Education, % (*n*)					
Low	31.9 (1822)	31.0 (1651)	30.9 (1366)	30.4 (1208)	20.5 (382)
Medium	37.7 (2156)	38.0 (2025)	35.1 (1551)	35.2 (1400)	32.4 (603)
High	30.4 (1741)	31.0 (1655)	33.9 (1499)	34.4 (1368)	47.1 (878)
Multimorbidity, % (*n*)	51.0 (2784)	55.7 (2856)	56.1 (2396)	57.7 (2147)	58.9 (1099)
Cognition, % (*n*)					
Low	29.9 (1660)	31.4 (1475)	30.8 (1212)	36.6 (1201)	37.1 (538)
Medium	41.7 (2319)	38.7 (1817)	38.0 (1498)	39.5 (1298)	31.9 (462)
High	28.4 (1579)	29.8 (1399)	31.2 (1230)	23.9 (786)	31.0 (449)
Smokers, % (*n*)	8.5 (488)	7.7 (396)	6.6 (282)	6.0 (224)	7.0 (130)
Alcohol drinkers, % (*n*)	44.5 (2545)	45.3 (2359)	40.6 (1738)	41.7 (1574)	37.8 (705)
Energy intake, *z*-score (mean ± SD)	−0.0 ± 1.0	−0.0 ± 1.0	−0.0 ± 1.0	−0.0 ± 1.0	−0.0 ± 1.0
Protein intake, g/kg BW/d, % (*n*)					
<0.8	33.6 (1924)	34.0 (1752)	36.8 (1566)	36.7 (1373)	45.5 (848)
0.8–0.99	23.3 (1332)	23.2 (1198)	22.9 (976)	22.4 (838)	21.2 (395)
1.0–1.19	19.3 (1107)	19.0 (981)	18.2 (773)	18.5 (691)	15.7 (292)
≥1.2	23.8 (1362)	23.8 (1227)	22.1 (942)	22.5 (842)	17.7 (330)
Protein intake, g/kg aBW/d, % (*n*)					
<0.8	27.6 (1579)	28.1 (1427)	30.4 (1293)	30.0 (1117)	39.4 (734)
0.8–0.99	23.3 (1335)	23.1 (1173)	24.5 (1043)	23.7 (881)	22.6 (421)
1.0–1.19	21.3 (1218)	20.8 (1056)	19.7 (836)	20.9 (777)	17.4 (324)
≥1.2	27.8 (1593)	27.9 (1415)	25.4 (1078)	25.5 (948)	20.7 (386)
Physical activity, % (*n*)					
Low	32.3 (1849)	37.9 (1933)	44.8 (1848)	43.7 (1541)	51.0 (870)
Medium	33.8 (1935)	39.7 (2025)	31.2 (1287)	32.0 (1127)	30.1 (514)
High	33.9 (1937)	22.5 (1146)	24.1 (993)	24.3 (856)	18.9 (322)
Walking speed, m/s (mean ± SD)	1.06 ± 0.28	1.06 ± 0.29	1.03 ± 0.27	1.01 ± 0.25	1.03 ± 0.23
Walking speed, *z*-score (mean ± SD)	−0.00 ± 1.00	−0.02 ± 1.00	−0.14 ± 1.05	−0.28 ± 1.06	−0.53 ± 1.08
Mobility limitation, % (*n*)					
Difficulty walking >200 m	18.0 (1031)	21.2 (1048)	25.2 (981)	30.2 (1092)	35.4 (605)
Difficulty climbing stairs	21.3 (1208)	24.2 (1233)	30.0 (1246)	31.5 (1129)	25.5 (414)

1 Not all waves are at the same time of follow-up among cohorts. In Health ABC the necessary variables were available at year 2 (operationalized as baseline), 4 (wave 2), 6 (wave 3), 8 (wave 4), and 10 (wave 5). In NuAge data were available at year 1 (baseline), 2 (wave 2), 3 (wave 3), and 4 (wave 4). In LASA variables were available at wave 3B (baseline) and at wave I after 3 y (wave 2). The Newcastle 85+ has data at baseline, after 18 mo (wave 2), after 36 mo (wave 3), and after 60 mo (wave 4). Cognition was assessed with the Mini-Mental State Examination. Smokers and alcohol drinkers represent current consumers. *z*-scores and tertiles are cohort-specific. aBW, adjusted body weight; BW, body weight; Health ABC, Health, Aging and Body Composition Study; LASA, Longitudinal Aging Study Amsterdam; N85+, Newcastle 85+ Study; NuAge, Quebec Longitudinal Study on Nutrition and Successful Aging.

### Protein intake and walking speed

**FIGURE 2:**
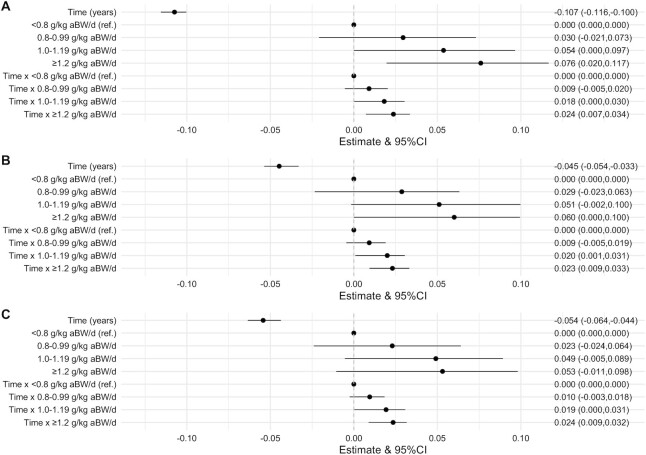
Association between protein intake categories (g/kg aBW/d) and walking speed (*z*-score) over time (β coefficients and 95% CIs). The analytic sample consisted of 5725 participants. A joint model (hierarchical linear mixed effects and Cox proportional hazards models) was fitted to assess the association between protein intake and walking speed over time. Model 1 (A) is adjusted for categories of adjusted protein intake, sex, age, and education. Model 2 (B) is further adjusted for energy, smoking, and alcohol intake, and Model 3 (C) is further adjusted for cognition, multimorbidity, and physical activity. aBW, adjusted body weight.

In the model adjusted for sex, age, and education, walking speed (*z*-score) decreased on average by 0.107 SD (95% CI: −0.116, −0.100) per year. In the same model, higher protein intake was modestly associated with faster walking speed and less decline in walking speed over time (**Figure 2**A). For example, a protein intake ≥1.2 g/kg aBW/d was associated with 0.076 SD (95% CI: 0.020, 0.117) faster walking speed and 0.024 SD (95% CI: 0.007, 0.034) per year slower decline in walking speed compared with a protein intake <0.8 g/kg aBW/d. There was also evidence of a dose-dependent association between protein intake category and decline in walking speed. The same trends remained with further adjustment for confounding factors (e.g., energy intake and multimorbidity) (Figure 2B, C). For example, in the fully adjusted model, a protein intake ≥1.2 compared with <0.8 g/kg aBW/d was associated with 0.053 SD (95% CI: −0.011, 0.098) faster walking speed and 0.024 SD (95% CI: 0.009, 0.032) per year slower decline in walking speed (Figure 2C). Results were similar for protein g/kg actual BW/d (**Supplemental Table 5**).

### Protein intake and mobility limitation

There were 1478 incident cases (i.e., transition from no limitation to mobility limitation) of difficulty walking >200 m and 742 who recovered (**Table 3**). There were also 1612 incident cases of difficulty climbing stairs and 932 who recovered (**Table 4**) from one wave to another (mean: 1.70 ± 0.55 y between each wave). In the fully adjusted models, higher protein intake was associated with a lower incidence of mobility limitation (for both difficulty walking >200 m and difficulty climbing stairs). Participants with a protein intake of 0.8–0.99 g/kg aBW/d were 16% less likely to develop difficulty walking (HR: 0.84; 95% CI: 0.72, 0.99) (Table 3) and 22% less likely to develop difficulty climbing stairs (HR: 0.78; 95% CI: 0.67, 0.92) (Table 4) compared with participants with protein intake <0.8 g/kg aBW/d. This protective association was also observed for a protein intake of 1.0–1.19 g/kg aBW/d (difficulty walking, HR: 0.71; 95% CI: 0.59, 0.86; difficulty climbing stairs, HR: 0.76; 95% CI: 0.63, 0.91), and of ≥1.2 g/kg aBW/d (difficulty walking, HR: 0.69; 95% CI: 0.56, 0.84; difficulty climbing stairs, HR: 0.76; 95% CI: 0.62, 0.92) compared with <0.8 g/kg aBW/d. Higher protein intake, even in participants with a protein intake ≥0.8 g/kg aBW/d, seemed to be beneficial for lower incidence of difficulty walking (Table 3), but not for difficulty climbing stairs, where all protein intake categories >0.8 cutoff had similar HRs (Table 4). No associations between protein intake and other transitions of mobility limitation were observed. Results were similar for protein in g/kg actual BW/d (**Supplemental Tables 6** and **7**).

**TABLE 3:** HRs and 95% CIs for the contribution of protein intake categories to transitions in self-reported difficulty walking^1^

	Protein intake, g/kg aBW/d						
	<0.8 (ref.)	0.8–0.99		1.0–1.19		≥1.2	
	HR	HR	95% CI	HR	95% CI	HR	95% CI
	Incident mobility limitation (*n* = 1478)						
Model 1	1.0	0.90	0.79, 1.04	0.82	0.71, 0.96	0.83	0.72, 0.95
Model 2	1.0	0.83	0.72, 0.97	0.72	0.60, 0.85	0.68	0.56, 0.82
Model 3	1.0	0.84	0.72, 0.99	0.71	0.59, 0.86	0.69	0.56, 0.84
	No mobility limitation to death (*n* = 542)						
Model 1	1.0	1.07	0.71, 1.59	1.24	0.84, 1.84	1.20	0.82, 1.76
Model 2	1.0	1.02	0.68, 1.52	1.13	0.74, 1.73	1.00	0.61, 1.64
Model 3	1.0	1.04	0.69, 1.56	1.20	0.78, 1.85	1.08	0.66, 1.78
	Recovery from mobility limitation (*n* = 742)						
Model 1	1.0	0.98	0.79, 1.20	0.98	0.79, 1.23	1.05	0.85, 1.29
Model 2	1.0	1.03	0.82, 1.30	1.04	0.81, 1.34	1.10	0.82, 1.48
Model 3	1.0	1.03	0.81, 1.32	1.06	0.81, 1.39	1.09	0.80, 1.48
	Mobility limitation to death (*n* = 557)						
Model 1	1.0	0.98	0.82, 1.16	0.98	0.81, 1.19	0.98	0.81, 1.17
Model 2	1.0	1.06	0.88, 1.27	1.10	0.89, 1.36	1.16	0.92, 1.46
Model 3	1.0	1.10	0.91, 1.33	1.13	0.91, 1.41	1.20	0.95, 1.52

1 Multistate models were used to determine the association between protein intake and transitions in difficulty walking. Model 1 is adjusted for categories of adjusted protein intake, sex, age, and education. Model 2 is further adjusted for energy intake, smoking, and alcohol intake, and Model 3 is further adjusted for cognition, multimorbidity, and physical activity. aBW, adjusted body weight; ref., referent.

**TABLE 4:** HRs and 95% CIs for the contribution of protein intake categories to transitions in self-reported difficulty climbing stairs^1^

	Protein intake, g/kg aBW/d						
	<0.8 (ref.)	0.8–0.99		1.0–1.19		≥1.2	
	HR	HR	95% CI	HR	95% CI	HR	95% CI
	Incident mobility limitation (*n* = 1612)						
Model 1	1.0	0.83	0.72, 0.96	0.83	0.72, 0.97	0.82	0.71, 0.95
Model 2	1.0	0.79	0.68, 0.92	0.74	0.62, 0.88	0.70	0.58, 0.85
Model 3	1.0	0.78	0.67, 0.92	0.76	0.63, 0.91	0.76	0.62, 0.92
	No mobility limitation to death (*n* = 608)						
Model 1	1.0	1.18	0.84, 1.66	1.27	0.89, 1.81	1.13	0.80, 1.61
Model 2	1.0	1.16	0.81, 1.66	1.28	0.86, 1.91	1.07	0.67, 1.71
Model 3	1.0	1.16	0.83, 1.61	1.23	0.85, 1.79	1.12	0.73, 1.72
	Recovery from mobility limitation (*n* = 932)						
Model 1	1.0	0.88	0.73, 1.07	0.84	0.68, 1.03	0.91	0.75, 1.10
Model 2	1.0	0.89	0.73, 1.09	0.86	0.68, 1.08	0.93	0.71, 1.20
Model 3	1.0	0.91	0.73, 1.13	0.92	0.72, 1.18	1.05	0.80, 1.38
	Mobility limitation to death (*n* = 598)						
Model 1	1.0	0.89	0.75, 1.06	0.96	0.79, 1.17	0.92	0.77, 1.10
Model 2	1.0	0.98	0.81, 1.18	1.03	0.83, 1.28	1.07	0.85, 1.34
Model 3	1.0	1.01	0.83, 1.23	1.06	0.84, 1.33	1.12	0.88, 1.43

1 Multistate models were used to determine the association between protein intake and transitions in difficulty climbing stairs. Model 1 is adjusted for categories of adjusted protein intake, sex, age, and education. Model 2 is further adjusted for energy intake, smoking, and alcohol intake, and Model 3 is further adjusted for cognition, multimorbidity, and physical activity. aBW, adjusted body weight; ref., referent.

### Interaction of protein intake with PA

**FIGURE 3:**
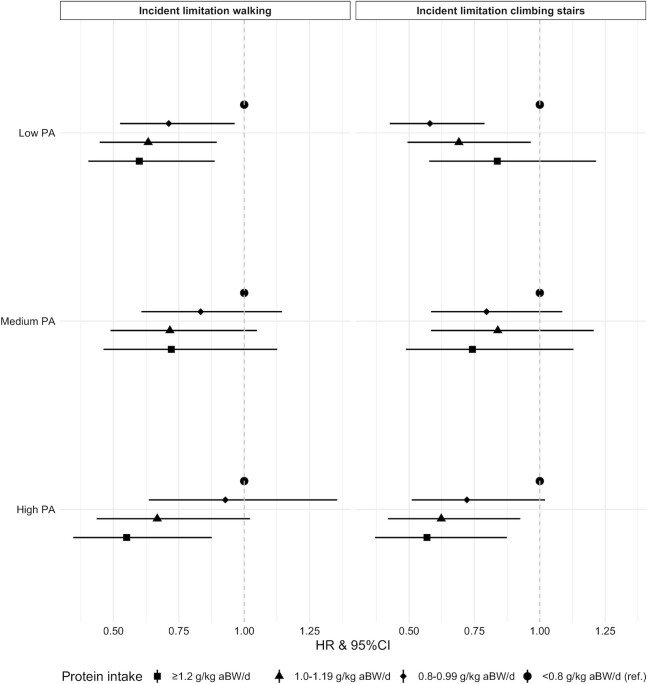
HRs and 95% CIs for the contribution of protein intake in grams per kilogram adjusted body weight per day to incident mobility limitations (i.e., difficulty walking >200 m and climbing stairs) by physical activity category over time. The analytic sample for the incident limitation climbing stairs model consisted of 1612 transitions, and for the incident limitation walking model consisted of 1478 transitions. Multistate models were used to determine the association between protein intake and transitions to mobility limitation stratified by physical activity. The models are adjusted for categories of adjusted protein intake, sex, age, and education, energy intake, smoking, alcohol intake, cognition, and multimorbidity and stratified by PA category at baseline. aBW, adjusted body weight; PA, physical activity.

The fully adjusted model for protein intake and walking speed was stratified by low, medium, and high PA (**Supplemental Figure 3**). Although there was a trend within the same PA category for a slightly slower decline in walking speed for higher protein intake [e.g., participants with ≥1.2 compared with <0.8 g protein/kg aBW/d and medium PA had a slower decline in walking speed of 0.03 SD (95% CI: 0, 0.05)/y], there was no evidence of other associations (Supplemental Figure 3). No interactions between protein intake and PA were observed for any of the transitions between mobility limitation (i.e., difficulty walking and climbing stairs), except for incident mobility limitation. For the latter, we ran the same fully adjusted models by PA category (**Figure 3**) and observed that higher protein intake was associated with a decreased likelihood of transitioning from no mobility limitation to mobility limitation for both difficulty walking and difficulty climbing stairs, and within low PA and high PA but not within medium PA. These associations seemed to be largely dose-dependent, where participants with higher protein intake had a lower likelihood of incident mobility limitation, even within the same PA category. For example, participants with a protein intake ≥1.2 g/kg aBW/d and high PA were 43% less likely (HR: 0.57; 95% CI: 0.37, 0.87) than those with a protein intake <0.8 g/kg aBW/d and high PA to transition to difficulty climbing stairs, whereas participants with a protein intake of 1.0–1.19 g/kg aBW/d were 38% less likely (HR: 0.62; 95% CI: 0.42, 0.93) and those with a protein intake of 0.8–0.99 g/kg aBW/d were 28% less likely (HR: 0.72; 95% CI: 0.51, 1.02) to transition to difficulty climbing stairs within the high PA category (Figure 3).

## Discussion

To the best of our knowledge, we are the first to perform a pooled analysis of individual participant data from multiple longitudinal aging cohorts on the prospective association between protein intake and (objective and subjective) physical function, and its interaction with PA. Our study indicated that community-dwelling older adults with protein intake higher than the EFSA's and IoM's RDA of 0.8 g/kg BW/d were more likely to retain objective and perceived physical function over time. Those with protein intake ≥0.8 g/kg aBW/d had (moderate) slower decline in walking speed and were less likely to develop self-reported mobility limitation, with some evidence for dose-dependency. Our study also indicated that a protein intake ≥1.0 g/kg aBW/d was associated with less functional decline compared with an intake of 0.8–0.99 g/kg aBW/d. We observed no synergistic relation between protein intake and PA for maintaining physical function over time.

Our finding that higher protein intake can be associated with a modest decline in walking speed over a follow-up of maximum 8.5 y is in contrast to the individual studies pooled (9, 13, 15), and to other aging cohorts (12, 14), except one (11). This could be explained by the larger sample size in our study, providing more power to detect this modest association, especially because the decline in walking speed was small (prior to *z*-score transformation it declined by 0.05 m/s over the study period), which could be insufficient to see a more clinically relevant association with higher protein intake. Our study mostly comprised well-functioning older adults at baseline, among whom the rate of functional decline might be slower than those who are frailer and/or have more comorbidities (59). Two recent meta-analyses of RCTs of protein supplementation and physical performance, in nonfrail and frail/ill older adults (22, 60), support this. That in nonfrail older adults failed to show an effect of protein supplementation on walking speed (22), whereas that in frail and ill older adults showed that protein supplementation improved physical function (60).

Regarding protein intake and mobility limitations, our finding that older adults who consumed ≥0.8 g protein/kg aBW/d were less likely to develop mobility limitations, compared with those with a protein intake <0.8 g/kg aBW/d, was in line with previous studies in individual cohorts used in this study (10, 61) and in other aging cohorts (12, 62–64). We observed no associations between protein intake and other mobility limitation transitions, that is, from no mobility limitation to death, recovery from mobility limitation, or from mobility limitation to death. These findings suggest that higher protein intake is especially beneficial in the prevention of mobility limitation rather than reversing it, because it might be too late when mobility limitation develops, and emphasize the importance of early screening for lower protein intake and early (dietary) intervention.

The debate about whether the RDA for protein for older adults, currently 0.8 g/kg BW/d according to the EFSA (35) and IoM (36), should be increased, and to what extent, is still ongoing. Our results tend to show that the relation between protein intake and decline in walking speed is dose-dependent. There was also a small reduction in the likelihood of incident difficulty walking with higher protein intake categories (0.8–0.99, 1.0–1.19, and ≥1.2 g/kg aBW/d) suggesting, as for walking speed, a dose-dependent association, although this was not observed for difficulty climbing stairs. These findings suggest that higher protein intake might benefit walking function not only in older adults with a protein intake below the RDA, but also in those with a habitual protein intake of ∼1.0 g/kg BW/d [mean protein intake in community-dwelling European and North American older adults (17)]. Our findings are in line with results from the study by Beasley et al. (12), in which the rate of self-reported physical function decline was 52% lower in females with a mean protein intake of 1.19 ± 0.20 g/kg BW/d than in females with a protein intake of 0.97 ± 0.17 g/kg BW/d. Together, these results suggest that even a protein intake >1.2 g/kg BW/d might be better in terms of slowing the rate of physical function decline. However, as such a (high) protein recommendation could have many substantial implications (e.g., for >70% of community-dwelling older adults, health care, food industry, and environment), such recommendations should only be set when sufficient high-quality evidence is available. More observational studies and RCTs in well-characterized study samples with adequate follow-up are needed to define the optimal yet feasible protein intake in older adults as well as the optimal food sources for the additional protein.

Several experts have suggested that (higher) PA could have an additive or synergistic effect on muscle and physical function when combined with protein intake (18). We did not find a clear indication for effect modification by PA or a synergistic effect of protein and PA. We observed a trend of higher protein intake categories being more protective for walking speed and walking speed decline within both the low and high PA category, but these did not reach statistical significance. This is probably a consequence of the small decline in walking speed over time. Participants with higher protein intake (even ≥0.8 g/kg aBW/d) were less likely to transition to mobility limitation, within each PA level. This suggests that *1*) higher protein intake can protect from the incidence of mobility limitation independently of PA level, and *2*) participants with protein intake ≥0.8 g/kg aBW/d still benefit from higher protein intake within the same PA level (i.e., a protein intake ≥1.2 g/kg aBW/d has a greater protective association with incident mobility limitations than 0.8–0.99 g/kg aBW/d). Similar to our findings, 1 systematic review of 15 RCTs in older adults on the additive effect of protein supplementation and progressive resistance exercise for 2–5 times/wk over 7 wk to 1 y found that improvements in physical function were no different with protein supplementation alone or in combination with resistance exercise (23). A later systematic review of 36 RCTs in nonfrail older adults concluded that walking speed tended to improve more with protein supplementation in combination with resistance exercise after 26 ± 26 wk compared with resistance exercise only (22). Differences in the type, intensity, and duration of PA or the timing in relation to protein intake might account for different effects of PA (and its interaction with protein) on physical function.

A major strength of this study is that we harmonized data from 4 large aging cohorts and performed an individual participant pooled analysis, allowing us to significantly increase our sample size and test for interactions that we could not test otherwise. The use of objective (walking speed) and subjective (mobility limitations) measures of physical function, the large range of covariates adjusted for, and the use of joint modeling and multistate models to account for nonrandom attrition and study membership, are other major strengths of this study.

One possibly important limitation in our study is misreporting of dietary intakes. However, because protein-rich foods are less commonly underreported (unlike snacks and sweets) (65) it is unlikely that protein intakes were underestimated. Additionally, although protein intake was harmonized, dietary intake was assessed by FFQs in Health ABC and LASA, and with multiple 24-h recalls in NuAge and Newcastle 85+, 2 methods that might give slightly different estimates. Another limitation is that protein intake was measured at baseline only and, therefore, intakes were assumed to be stable or have declined proportionally over the follow-up period. A fourth limitation is that mobility limitation states were assigned at every data collection point (mean =1.70 ± 0.55 y) between each wave but unobserved incidence and recovery from these mobility limitation states could have occurred between waves. Finally, although attempts were made to reduce the possibility of reverse causality it is impossible to fully exclude the possibility of protein intake being affected by physical function.

In conclusion, increasing daily protein intake can reduce physical function decline over 8.5 y not only in community-dwelling older adults with a protein intake at or below the current RDA of 0.8 g/kg BW/d, but also in those with a protein intake that is already considered sufficient. This dose-dependent association was observed for each level of PA, suggesting no clear synergistic association between protein intake and PA in relation to physical function.

## Supplementary Material

nqab051_Supplemental_File

## Notes

Funding for this research is provided by the European Horizon 2020 PROMISS Project “PRevention Of Malnutrition In Senior Subjects in the EU,” grant agreement no. 678732. NM has received funding from the European Union's Horizon 2020 research and innovation program under the Marie Sklodowska-Curie grant agreement no. 835577. Health ABC has been supported by the Intramural Research Program of the NIH, National Institute on Aging (NIA) (NIA contracts N01-AG-6-2101, N01-AG-6-2103, and N01-AG-6-2106; NIA grant R01-AG028050; National Institute of Nursing Research grant R01-NR012459). The NuAge study was funded by the Canadian Institutes of Health Research (CIHR; MOP-62842). The NuAge Database and Biobank are supported by the Fonds de recherche du Québec (FRQ; 2020-VICO-279753), the Quebec Network for Research on Aging, a thematic network funded by the Fonds de Recherche du Québec—Santé (FRQS), and by the Merck-Frosst Chair funded by La Fondation de l'Université de Sherbrooke. NP is a Junior 1 Research Scholar of the FRQS. The Longitudinal Aging Study Amsterdam is supported by a grant from the Netherlands Ministry of Health, Welfare and Sports, Directorate of Long-Term Care. The Newcastle 85+ Study has been funded by a joint grant from the Medical Research Council and Biotechnology and Biological Sciences Research Council (G0500997), and the Dunhill Medical Trust (R124/0509).

The content reflects the authors’ views only and the European Commission is not responsible for any use made of the information the article contains.

The data described in the manuscript, code book, and analytic code will be made available upon request pending application and approval from individual cohorts, and payment for management costs.

Supplemental Methods, Supplemental Tables 1–7, and Supplemental Figures 1–3 are available from the “Supplementary data” link in the online posting of the article and from the same link in the online table of contents at https://academic.oup.com/ajcn/.

Abbreviations used: aBW, adjusted body weight; BW, body weight; Health ABC, the Health Aging and Body Composition Study; EFSA, European Food Safety Authority; HLY, healthy life year; IoM, Institute of Medicine; LASA, Longitudinal Aging Study Amsterdam; MET, metabolic equivalent; Miss, missing; NuAge, Quebec Longitudinal Study on Nutrition and Successful Aging; PA, physical activity; PROMISS, PRevention Of Malnutrition In Senior Subjects in the EU; RCT, randomized controlled trial; TUG, Timed-Up-and-Go.
