# Supplementary material for: Low protein intake, physical activity, and physical function in European and North American community-dwelling older adults: a pooled analysis of four longitudinal aging cohorts
Source: Am J Clin Nutr. 2021 Apr 7;114(1):29–41. doi: 10.1093/ajcn/nqab051 (PMC8246618; doi:10.1093/ajcn/nqab051)
Supplement: nqab051_Supplemental_File [file nqab051_supplemental_file.docx]

**On-line Supplementary Material**

***Low protein intake, physical activity and physical function in European and North American community-dwelling older adults: a pooled analysis of four longitudinal aging cohorts***

Nuno Mendonça ^1-3*^, Linda M. Hengeveld ^4^, Marjolein Visser ^4^, Nancy Presse ^5-7^, Helena Canhão ^1,2^, Eleanor M. Simonsick ^8^, Stephen B. Kritchevsky ^9^, Anne B. Newman ^10^, Pierrette Gaudreau ^11^, Carol Jagger ^3^

^1^ EpiDoC Unit, NOVA Medical School, Universidade Nova de Lisboa (NMS-UNL), Lisbon, Portugal, ^2^ Comprehensive Health Research Centre (CHRC), Portugal, ^3^ Population Health Sciences Institute, Newcastle University, Newcastle-upon-Tyne, UK, ^4^ Department of Health Sciences, Faculty of Science, Amsterdam Public Health research institute, Vrije Universiteit Amsterdam, Amsterdam, the Netherlands, ^5^ Research Centre on Aging, CIUSSS de l’Estrie-CHUS, Sherbrooke, Quebec, Canada, ^6^ Faculty of Medicine and Health Sciences, University of Sherbrooke, Sherbrooke, Quebec, Canada,^7^ Centre de recherche de l’Institut universitaire de gériatrie de Montréal, Montréal, Quebec, Canada,^8^ National Institute on Aging Intramural Research Program, Baltimore, Maryland, USA, ^9^ Sticht Center on Aging, Wake Forest School of Medicine, Winston-Salem, North Carolina, USA, ^10^ Center for Aging and Population Health, Department of Epidemiology, University of Pittsburgh, Pennsylvania, USA, ^11^ Department of Medicine, University of Montreal and Research Centre of the University of Montreal Hospital Centre, Montreal, Quebec, Canada.

*****Corresponding author: Nuno Mendonça, address: NOVA Medical School, Universidade Nova de Lisboa, Rua do Instituto Bacteriológico, nº5, 1150-082 Lisboa, Portugal, email: nuno.mendonca@nms.unl.pt, telephone: +351218803110

**Supplementary Methods**

*Education, BMI, Smoking, Alcohol drinking, Multimorbidity and Cognitive status*

Education level was based on the self-reported highest level of education attained (Health ABC, LASA) or years of full time education (Newcastle 85+, NuAge) and categorized into: low (finished lower vocational education, middle school or lower; or ≤9 years of education), medium (finished general intermediate or attended (but not finished) higher vocational education or high school; or 10 to 14 years of education) and high (finished higher vocational education or university; or ≥15 years of education). BMI, calculated as body weight (kg) divided by height (m) squared, was based on measured body weight and measured body height in all studies except in Newcastle 85+ where height was calculated from measured demi-span. In all four cohorts smoking was defined as currently smoking. In Health ABC and LASA, alcohol drinkers were defined as having reported drinking alcohol in the FFQ, and in NuAge and Newcastle 85+ from the multiple 24-h recalls. Cognitive function in Health ABC and NuAge was assessed by the Teng 3MS (extension of the Mini-Mental State Examination (MMSE) with a 100-point scale) (1), with the MMSE scores for these two cohorts being computed from the Teng 3MS. In LASA and Newcastle 85+, cognitive function was assessed with the MMSE (2). The MMSE score was then transformed into cohort-specific tertiles (categorized as low, medium and high cognitive function) at baseline which were used to categorize the MMSE score for the following waves. Disease burden in Health ABC was determined by creating a disease score from self-reported cancer (except non-melanoma skin cancer), cardiac diseases (congestive heart disease and myocardial infarction), cerebrovascular diseases (stroke), hypertension (systolic blood pressure ≥130 mmHg and diastolic blood pressure ≥85 mmg Hg) and diabetes mellitus (fasting blood glucose ≥126 mg/dl). In NuAge, a disease score was created by summing the presence of self-reported respiratory diseases (asthma, emphysema, chronic bronchitis), arthritis or rheumatism, hypertension, cardiac diseases, diabetes mellitus, transient ischemic attack, stroke and cancer. In LASA, the 7 most common chronic diseases in the Netherlands were assessed by asking about the presence of lung disease, cardiac disease, peripheral arterial disease, diabetes mellitus, stroke, osteoarthritis or rheumatoid arthritis and cancer (except non-melanoma skin cancer) plus hypertension. Lastly, in Newcastle 85+, general practitioner records were reviewed for the presence or absence of cardiac disease, arthritis, hypertension, respiratory disease, cerebrovascular disease, diabetes mellitus and cancer (excluding non-melanoma skin cancer). A cohort-specific disease score of 2 or more was considered multimorbidity. Education was only available at baseline and assumed to remain the same throughout follow-up. Alcohol drinking was only available at baseline in all cohorts except for NuAge where it was available for every wave. Baseline smoking was used for Health ABC and Newcastle 85+, but for all waves in NuAge and LASA. BMI, cognitive function and multimorbidity were available at every wave.

**Supplementary Table 1.** Baseline health and socio-demographic characteristics of participants by having or not having data on walking speed (WS) and self-reported mobility limitation (i.e. difficulty walking >200m or difficulty climbing stairs).

|  | **No WS** (n=52) | **WS** (n=5673) | *p* | **No Stairs/Walk** (n=51) | **Stairs/Walk** (n=5674) | *p* |
| --- | --- | --- | --- | --- | --- | --- |
| Age, y (median, IQR) | 75.0 [71.0-78.2] | 75.0 [71.6-79.0] | 0.997 | 75.0 [73.0-78.0] | 75.0 [71.6-79.0] | 0.482 |
| Females % (n) | 57.7 (30) | 53.0 (3005) | 0.589 | 66.7 (34) | 52.9 (3001) | 0.069 |
| Cohort % (n) |  |  | 0.005 |  |  | <0.001 |
| Health ABC | 61.5 (32) | 46.3 (2628) |  | 92.2 (47) | 46.1 (2613) |  |
| NuAge | 7.7 (4) | 30.4 (1722) |  | 7.8 (4) | 30.3 (1722) |  |
| LASA | 15.4 (8) | 10.8 (612) |  | 0.0 (0) | 10.9 (620) |  |
| N85+ | 15.4 (8) | 12.5 (711) |  | 0.0 (0) | 12.7 (719) |  |
| Education % (n) |  |  | 0.415 |  |  | 0.010 |
| Low | 40.4 (21) | 31.8 (1801) |  | 51.0 (26) | 31.7 (1796) |  |
| Medium | 32.7 (17) | 37.7 (2139) |  | 31.4 (16) | 37.8 (2140) |  |
| High | 26.9 (14) | 30.5 (1727) |  | 17.6 (9) | 30.6 (1732) |  |
| Multimorbidity % (n) | 61.5 (32) | 50.9 (2752) | 0.165 | 56.9 (29) | 50.9 (2755) | 0.483 |
| Cognition % (n) |  |  | 0.629 |  |  | <0.001 |
| Low | 34.6 (18) | 29.8 (1642) |  | 61.2 (30) | 29.6 (1630) |  |
| Medium | 42.3 (22) | 41.7 (2297) |  | 16.3 (8) | 41.9 (2311) |  |
| High | 23.1 (12) | 28.5 (1567) |  | 22.4 (11) | 28.5 (1568) |  |
| Smokers % (n) | 19.6 (10) | 8.4 (478) | 0.010 | 14.0 (7) | 8.5 (481) | 0.258 |
| BMI (mean ± SD) | 27.2 (4.7) | 27.0 (4.8) | 0.821 | 28.9 (6.5) | 27.0 (4.7) | 0.005 |
| Alcohol drinkers % (n) | 34.6 (18) | 44.5 (2527) | 0.196 | 15.7 (8) | 44.7 (2537) | <0.001 |
| Energy intake, z-score (mean ± SD) | -0.3 (0.9) | 0.0 (1.0) | 0.053 | -0.0 (1.2) | 0.0 (1.0) | 0.817 |
| Physical Activity % (n) |  |  | 0.005 |  |  | <0.001 |
| Low | 52.9 (27) | 32.1 (1822) |  | 66.7 (34) | 32.0 (1815) |  |
| Medium | 27.5 (14) | 33.9 (1921) |  | 21.6 (11) | 33.9 (1924) |  |
| High | 19.6 (10) | 34.0 (1927) |  | 11.8 (6) | 34.1 (1931) |  |
| Walking speed, z-score (mean ± SD) | - | -0.0 (1.0) | - | -0.8 (0.9) | 0.0 (1.0) | <0.001 |
| Mobility limitation % (n) |  |  |  |  |  |  |
| Difficulty walking >200m | 44.2 (23) | 17.8 (1008) | <0.001 | 45.1 (23) | 17.8 (1008) | <0.001 |
| Difficulty climbing stairs | 43.1 (22) | 21.1 (1186) | <0.001 | - | 21.3 (1208) | - |

Cognition was assessed with the Mini-Mental State Examination. Smokers and alcohol drinkers represent current consumers. The number of participants without the data for self-reported difficulty climbing stairs is the same as those who do not have data for difficulty walking >200m. z-scores and tertiles are cohort-specific. Non-difference between missing and non-missing walking speed or mobility limitations was assessed with chi-squared test (χ2) for categorical variables and independent samples t-test/ Mann-Whitney U test for continuous variables along with the effect size and, SD or 95% CI. aBW, adjusted body weight; BMI, body mass index; Health ABC, Health, Aging and Body Composition Study; IQR, interquartile range; LASA, Longitudinal Aging Study Amsterdam; MMSE, Mini-Mental State Examination; N85+, Newcastle 85+ Study; NuAge, Quebec Longitudinal Study on Nutrition and Successful Aging; SD, standard deviation; WS, walking speed; y, years.

**Supplementary Table 2.** Description of selected variables.

| **Variable** | **Description** | **Wave**^1^ | **Coding** |
| --- | --- | --- | --- |
| id | (Pseudo-)anonymous unique identifier | Baseline | string |
| Study | Cohort identifier | Baseline | 1=Health ABC  2=NuAge  3=LASA  4=Newcastle 85+ |
| **Exposure** | | | |
| Protein intake (non-adjusted) | Protein intake per non-adjusted body weight per day (g/kg BW/d) | Baseline | 0=<0.8 g/kg BW/d  1=0.8-0.99 g/kg BW/d  2=1.0-1.19 g/kg BW/d  3=≥1.2 g/kg BW/d |
| Protein intake (adjusted) | Protein intake per adjusted body weight per day (g/kg aBW/d) | Baseline | 0=<0.8 g/kg aBW/d  1=0.8-0.99 g/kg aBW/d  2=1.0-1.19 g/kg aBW/d  3=≥1.2 g/kg aBW/d |
| **Moderator** | | | |
| Sex | Self-reported biological sex | Baseline | 0=males  1=females |
| Physical activity | Cohort-specific tertiles of self-reported physical activity | Baseline, wave 2,3,4,5 | 0=low physical activity  1=medium physical activity  2=high physical activity |
| **Outcome** | | | |
| Walking speed | Cohort-specific z-scores of time (s) taken to go from A to B where the distance (m) is divided by time (s), yielding walking speed (m/s) | Baseline, wave 2,3,4,5 | numerical |
| Climbing stairs | Self-reported ability to climb stairs | Baseline, wave 2,3,4,5 | 0=able to alone  1=difficulty/unable/requires help |
| Walking | Self-reported ability to walk >200m | Baseline, wave 2,3,4,5 | 0=able to alone  1=difficulty/unable/requires help |
| Mortality | Death event | NA | 0=alive by censoring date  1=dead |
| Survival age | Age at death or censoring | Baseline, wave 2,3,4,5 | numerical |
| **Possible Confounders** | | | |
| Age | Age in years since date of birth | Baseline, wave 2,3,4,5 | numerical |
| Education | Self-reported highest level of education attained or total number of years of full-time education | Baseline | 0=low or ≤9 years  1= intermediate or 10-14 years  2=high or ≥15 years |
| Body mass index | Measured body weight (kg)/ height (m)^2^ | Baseline, wave 2,3,4,5 | numerical |
| Smokers | Whether participants currently smoke | Baseline, wave 2,3,4,5 | 0=non-smoker  1=smoker |
| Energy intake | Cohort-specific z-scores of total energy intake (MJ) per day | Baseline | numerical |
| Alcohol intake | Whether participants currently drink alcohol | Baseline, wave 2,3,4,5 | 0=non-drinker  1=drinker |
| Cognition | Cohort-specific tertiles of Mini-Mental State Examination (MMSE) | Baseline, wave 2,3,4,5 | 0=low MMSE  1=medium MMSE  2=high MMSE |
| Multimorbidity | Co-occurrence of two or more chronic diseases | Baseline, wave 2,3,4,5 | 0=< 2 chronic diseases  1=≥ 2 chronic diseases |

^1^ Not all waves are at the same time of follow-up among cohorts. In Health ABC the necessary variables were available at year 2 (operationalized as baseline), 4 (wave 2), 6 (wave 3), 8 (wave 4) and 10 (wave 5). In NuAge data were available at year 1 (baseline), 2 (wave 2), 3 (wave 3) and 4 (wave 4). In LASA variables were available at wave 3B (baseline) and at wave I after 3 years (wave 2). Newcastle 85+ has data at baseline, after 18 months (wave 2), after 36 months (wave 3) and after 60 months (wave 4).

**Supplementary Table 3.** Baseline health and socio-demographic characteristics of participants by protein intake categories and cohort.

|  | **Health ABC** (n=2660) | | | | **NuAge** (n=1726) | | | | **LASA** (n=620) | | | | **N85+** (n=719) | | | |
| --- | --- | --- | --- | --- | --- | --- | --- | --- | --- | --- | --- | --- | --- | --- | --- | --- |
|  | **<0.8** (n=1044) | **0.8-0.99** (n=598) | **1.0-1.19** (n=458) | **≥1.2** (n=560) | **<0.8** (n=256) | **0.8-0.99** (n=434) | **1.0-1.19** (n=450) | **≥1.2** (n=586) | **<0.8** (n=79) | **0.8-0.99** (n=108) | **1.0-1.19** (n=167) | **≥1.2** (n=266) | **<0.8** (n=200) | **0.8-0.99** (n=195) | **1.0-1.19** (n=143) | **≥1.2** (n=181) |
| Age, y (median, IQR) | 74.0 [72.0-77.0] | 74.0 [72.0-77.0] | 75.0 [72.0-77.0] | 74.0 [72.0-77.0] | 76.0 [73.1-79.6] | 75.1 [71.7-79.0] | 75.5 [72.1-79.2] | 74.8 [71.7-79.0] | 61.2 [57.9-63.4] | 60.8 [58.3-63.7] | 60.8 [58.4-63.1] | 60.1 [57.9-62.9] | 85.6 [85.3-85.9] | 85.6 [85.3-85.9] | 85.5 [85.2-85.8] | 85.5 [85.1-85.8] |
| Females % (n) | 50.1 (523) | 51.8 (310) | 50.4 (231) | 55.0 (308) | 57.0 (146) | 50.5 (219) | 52.7 (237) | 51.2 (300) | 57.0 (45) | 55.6 (60) | 57.5 (96) | 48.5 (129) | 67.5 (135) | 65.6 (128) | 61.5 (88) | 44.2 (80) |
| Education % (n) |  |  |  |  |  |  |  |  |  |  |  |  |  |  |  |  |
| Low | 23.2 (241) | 20.6 (123) | 21.7 (99) | 25.4 (142) | 41.0 (105) | 35.0 (152) | 33.8 (152) | 34.3 (201) | 16.5 (13) | 26.9 (29) | 20.4 (34) | 27.1 (72) | 65.5 (131) | 71.3 (139) | 56.6 (81) | 59.7 (108) |
| Medium | 33.9 (353) | 35.1 (210) | 28.1 (128) | 30.4 (170) | 42.6 (109) | 38.2 (166) | 38.4 (173) | 39.4 (231) | 67.1 (53) | 57.4 (62) | 65.3 (109) | 62.4 (166) | 32.5 (65) | 24.6 (48) | 35.0 (50) | 34.8 (63) |
| High | 42.9 (446) | 44.3 (265) | 50.2 (229) | 44.3 (248) | 16.4 (42) | 26.7 (116) | 27.8 (125) | 26.3 (154) | 16.5 (13) | 15.7 (17) | 14.4 (24) | 10.5 (28) | 2.0 (4) | 4.1 (8) | 8.4 (12) | 5.5 (10) |
| Multimorbidity % (n) | 41.6 (434) | 45.8 (274) | 44.1 (202) | 41.8 (234) | 56.7 (122) | 50.3 (181) | 49.7 (186) | 46.4 (237) | 72.2 (57) | 68.5 (74) | 68.3 (114) | 60.2 (160) | 75.0 (150) | 68.7 (134) | 72.0 (103) | 67.4 (122) |
| Cognition % (n) |  |  |  |  |  |  |  |  |  |  |  |  |  |  |  |  |
| Low | 32.2 (314) | 29.5 (166) | 33.6 (146) | 34.7 (183) | 29.0 (74) | 29.0 (126) | 28.1 (126) | 26.6 (155) | 22.8 (18) | 29.6 (32) | 24.6 (41) | 20.3 (54) | 33.0 (66) | 32.8 (64) | 30.8 (44) | 28.2 (51) |
| Medium | 37.8 (369) | 36.8 (207) | 34.1 (148) | 35.5 (187) | 51.8 (132) | 47.9 (208) | 50.0 (224) | 54.2 (316) | 51.9 (41) | 50.9 (55) | 43.1 (72) | 55.3 (147) | 26.0 (52) | 31.8 (62) | 28.0 (40) | 32.6 (59) |
| High | 29.9 (292) | 33.7 (190) | 32.3 (140) | 29.8 (157) | 19.2 (49) | 23.0 (100) | 21.9 (98) | 19.2 (112) | 25.3 (20) | 19.4 (21) | 32.3 (54) | 24.4 (65) | 41.0 (82) | 35.4 (69) | 41.3 (59) | 39.2 (71) |
| Smokers % (n) | 8.3 (86) | 8.4 (50) | 9.4 (43) | 11.4 (64) | 7.4 (19) | 8.3 (36) | 6.9 (31) | 5.3 (31) | 14.1 (11) | 16.2 (17) | 13.9 (23) | 13.0 (34) | 5.5 (11) | 4.6 (9) | 5.6 (8) | 8.3 (15) |
| Alcohol drinkers % (n) | 34.2 (357) | 34.8 (208) | 40.4 (185) | 37.9 (212) | 41.0 (105) | 46.8 (203) | 49.3 (222) | 50.9 (298) | 75.9 (60) | 79.6 (86) | 84.4 (141) | 75.9 (202) | 37.0 (74) | 36.9 (72) | 28.7 (41) | 43.6 (79) |
| Energy intake, z-score (mean ± SD) | -0.8 (0.6) | -0.0 (0.7) | 0.4 (0.7) | 1.1 (0.9) | -0.9 (0.7) | -0.3 (0.8) | 0.0 (0.8) | 0.7 (1.0) | -1.2 (0.5) | -0.5 (0.6) | -0.2 (0.6) | 0.7 (0.9) | -0.8 (0.7) | -0.1 (0.7) | 0.2 (0.7) | 0.8 (1.0) |
| Protein intake (g/d) | 43.2 (11.5) | 63.1 (8.9) | 76.3 (10.8) | 100.7 (22.4) | 47.1 (8.4) | 62.3 (8.4) | 74.4 (10.2) | 94.5 (18.1) | 49.6 (8.8) | 66.3 (9.3) | 79.2 (10.7) | 103.1 (18.5) | 42.6 (9.0) | 57.2 (9.0) | 68.8 (8.4) | 92.2 (19.4) |
| Protein intake (g/kg aBW/d) | 0.6 (0.1) | 0.9 (0.1) | 1.1 (0.1) | 1.5 (0.3) | 0.7 (0.1) | 0.9 (0.1) | 1.1 (0.1) | 1.4 (0.2) | 0.7 (0.1) | 0.9 (0.1) | 1.1 (0.1) | 1.5 (0.2) | 0.7 (0.1) | 0.9 (0.1) | 1.1 (0.1) | 1.4 (0.3) |
| Physical Activity % (n) |  |  |  |  |  |  |  |  |  |  |  |  |  |  |  |  |
| Low | 37.3 (389) | 28.6 (171) | 34.7 (159) | 29.9 (167) | 37.9 (97) | 33.3 (144) | 33.8 (152) | 31.1 (182) | 44.3 (35) | 29.6 (32) | 32.5 (54) | 32.0 (85) | 29.5 (59) | 27.2 (53) | 22.4 (32) | 21.0 (38) |
| Medium | 32.4 (338) | 37.9 (226) | 31.2 (143) | 32.0 (179) | 36.3 (93) | 33.3 (144) | 34.2 (154) | 31.4 (184) | 39.2 (31) | 31.5 (34) | 37.3 (62) | 29.7 (79) | 38.5 (77) | 35.4 (69) | 44.1 (63) | 32.6 (59) |
| High | 30.4 (317) | 33.5 (200) | 34.1 (156) | 38.1 (213) | 25.8 (66) | 33.5 (145) | 32.0 (144) | 37.5 (220) | 16.5 (13) | 38.9 (42) | 30.1 (50) | 38.3 (102) | 32.0 (64) | 37.4 (73) | 33.6 (48) | 46.4 (84) |
| Walking speed, m/s (mean ± SD) | 1.1 (0.2) | 1.1 (0.2) | 1.1 (0.2) | 1.1 (0.2) | 1.0 (0.2) | 1.1 (0.2) | 1.0 (0.2) | 1.1 (0.2) | 1.1 (0.3) | 1.1 (0.3) | 1.2 (0.4) | 1.2 (0.3) | 0.7 (0.3) | 0.7 (0.3) | 0.7 (0.3) | 0.8 (0.3) |
| Walking speed, z-score (mean ± SD) | -0.0 (1.0) | 0.0 (1.0) | 0.0 (1.1) | 0.0 (1.0) | -0.1 (1.0) | 0.1 (1.0) | -0.0 (0.9) | 0.0 (1.0) | -0.2 (0.9) | -0.1 (0.9) | 0.0 (1.1) | 0.1 (1.0) | -0.1 (1.0) | -0.1 (0.9) | 0.0 (1.0) | 0.2 (1.0) |
| Mobility limitation % (n) |  |  |  |  |  |  |  |  |  |  |  |  |  |  |  |  |
| Difficulty walking >200m | 20.1 (210) | 15.7 (94) | 18.6 (85) | 18.6 (104) | 13.7 (35) | 11.8 (51) | 11.1 (50) | 8.7 (51) | 13.9 (11) | 3.7 (4) | 5.4 (9) | 5.3 (14) | 47.5 (95) | 45.6 (89) | 37.8 (54) | 41.4 (75) |
| Difficulty climbing stairs | 22.2 (227) | 19.0 (112) | 17.2 (78) | 21.1 (115) | 19.1 (49) | 15.9 (69) | 16.7 (75) | 14.7 (86) | 17.7 (14) | 13.9 (15) | 14.4 (24) | 10.9 (29) | 50.0 (100) | 46.2 (90) | 37.8 (54) | 39.2 (71) |

Cognition was assessed with the Mini-Mental State Examination. Smokers and alcohol drinkers represent current consumers. z-scores and tertiles are cohort-specific. aBW, adjusted body weight; Health ABC, Health, Aging and Body Composition Study; IQR, interquartile range; LASA, Longitudinal Aging Study Amsterdam; MMSE, Mini-Mental State Examination; N85+, Newcastle 85+ Study; NuAge, Quebec Longitudinal Study on Nutrition and Successful Aging; SD, standard deviation; y, years.

**Supplementary Table 4.** Baseline health and socio-demographic characteristics of participants by protein intake and physical activity categories.

| **Prot (g/kg aBW/d)** | **<0.8** (n=1579) | | | **0.8-0.99** (n=1333) | | | **1.0-1.19** (n=1217) | | | **≥1.2** (n=1592) | | |
| --- | --- | --- | --- | --- | --- | --- | --- | --- | --- | --- | --- | --- |
| **Physical Activity** | **Low** (n=580) | **Medium** (n=539) | **High** (n=460) | **Low** (n=400) | **Medium** (n=473) | **High** (n=460) | **Low** (n=397) | **Medium** (n=422) | **High** (n=398) | **Low** (n=472) | **Medium** (n=501) | **High** (n=619) |
| Age, y (median, IQR) | 75.0 [72.0-79.0] | 75.0 [72.0-79.0] | 75.0 [72.0-78.0] | 75.7 [72.5-80.0] | 75.0 [72.0-79.0] | 74.0 [71.2-78.6] | 75.0 [71.0-79.2] | 75.0 [71.0-79.9] | 74.8 [71.0-78.0] | 74.0 [70.8-79.0] | 75.0 [71.0-79.0] | 73.3 [70.5- 78.0] |
| Females % (n) | 60.9 (353) | 56.4 (304) | 41.7 (192) | 60.0 (240) | 56.2 (266) | 45.7 (210) | 57.7 (229) | 57.8 (244) | 45.0 (179) | 53.8 (254) | 55.7 (279) | 45.9 (284) |
| Cohort % (n) |  |  |  |  |  |  |  |  |  |  |  |  |
| Health ABC | 67.1 (389) | 62.7 (338) | 68.9 (317) | 42.8 (171) | 47.8 (226) | 43.5 (200) | 40.1 (159) | 33.9 (143) | 39.2 (156) | 35.4 (167) | 35.7 (179) | 34.4 (213) |
| NuAge | 16.7 (97) | 17.3 (93) | 14.3 (66) | 36.0 (144) | 30.4 (144) | 31.5 (145) | 38.3 (152) | 36.5 (154) | 36.2 (144) | 38.6 (182) | 36.7 (184) | 35.5 (220) |
| LASA | 6.0 (35) | 5.8 (31) | 2.8 (13) | 8.0 (32) | 7.2 (34) | 9.1 (42) | 13.6 (54) | 14.7 (62) | 12.6 (50) | 18.0 (85) | 15.8 (79) | 16.5 (102) |
| N85+ | 10.2 (59) | 14.3 (77) | 13.9 (64) | 13.2 (53) | 14.6 (69) | 15.9 (73) | 8.1 (32) | 14.9 (63) | 12.1 (48) | 8.1 (38) | 11.8 (59) | 13.6 (84) |
| Education % (n) |  |  |  |  |  |  |  |  |  |  |  |  |
| Low | 30.6 (177) | 30.7 (165) | 32.2 (148) | 33.5 (134) | 30.7 (145) | 35.4 (163) | 29.5 (117) | 30.3 (128) | 30.2 (120) | 32.4 (153) | 32.1 (161) | 33.8 (209) |
| Medium | 40.8 (236) | 36.7 (197) | 32.0 (147) | 38.2 (153) | 35.9 (170) | 35.4 (163) | 36.6 (145) | 38.6 (163) | 38.3 (152) | 41.1 (194) | 40.9 (205) | 37.2 (230) |
| High | 28.5 (165) | 32.6 (175) | 35.9 (165) | 28.2 (113) | 33.4 (158) | 29.1 (134) | 33.8 (134) | 31.0 (131) | 31.5 (125) | 26.5 (125) | 26.9 (135) | 29.1 (180) |
| Multimorbidity % (n) | 54.3 (306) | 48.7 (256) | 44.9 (201) | 60.2 (227) | 51.5 (229) | 47.1 (206) | 61.0 (227) | 53.9 (212) | 43.9 (165) | 54.6 (248) | 49.6 (237) | 45.6 (267) |
| Cognition % (n) |  |  |  |  |  |  |  |  |  |  |  |  |
| Low | 35.3 (195) | 31.0 (161) | 26.5 (116) | 29.9 (116) | 30.7 (141) | 29.1 (131) | 36.4 (143) | 26.4 (109) | 27.3 (105) | 33.8 (155) | 27.4 (133) | 25.3 (155) |
| Medium | 41.4 (229) | 38.2 (198) | 38.2 (167) | 45.4 (176) | 37.4 (172) | 40.7 (183) | 41.7 (164) | 40.7 (168) | 39.5 (152) | 44.1 (202) | 45.6 (221) | 46.5 (285) |
| High | 23.3 (129) | 30.8 (160) | 35.2 (154) | 24.7 (96) | 32.0 (147) | 30.2 (136) | 21.9 (86) | 32.9 (136) | 33.2 (128) | 22.1 (101) | 27.0 (131) | 28.2 (173) |
| Smokers % (n) | 12.0 (69) | 7.4 (40) | 3.9 (18) | 11.2 (45) | 6.6 (31) | 7.8 (36) | 12.9 (51) | 6.4 (27) | 6.8 (27) | 10.0 (47) | 9.0 (45) | 8.4 (52) |
| Alcohol drinkers % (n) | 32.8 (190) | 38.6 (208) | 43.0 (198) | 36.0 (144) | 46.1 (218) | 44.6 (205) | 46.3 (184) | 47.9 (202) | 51.0 (203) | 46.6 (220) | 52.5 (263) | 49.8 (308) |
| Energy intake, z-score (mean ± SD) | -0.9 (0.6) | -0.8 (0.6) | -0.7 (0.7) | -0.2 (0.7) | -0.2 (0.7) | -0.1 (0.7) | 0.1 (0.8) | 0.1 (0.7) | 0.3 (0.8) | 0.7 (0.9) | 0.8 (0.9) | 1.0 (1.0) |
| Walking speed, z-score (mean ± SD) | -0.4 (0.9) | -0.1 (0.9) | 0.4 (0.9) | -0.4 (1.0) | 0.0 (0.9) | 0.3 (0.9) | -0.3 (1.0) | -0.0 (1.0) | 0.3 (0.9) | -0.3 (1.0) | 0.1 (1.0) | 0.3 (0.9) |
| Mobility limitations |  |  |  |  |  |  |  |  |  |  |  |  |
| Difficulty walking >200m | 34.1 (198) | 20.6 (111) | 9.1 (42) | 30.1 (120) | 16.7 (79) | 8.5 (39) | 27.8 (110) | 14.7 (62) | 6.3 (25) | 26.1 (123) | 15.8 (79) | 6.8 (42) |
| climbing stairs | 34.2 (193) | 24.4 (131) | 14.4 (66) | 33.4 (132) | 19.4 (91) | 13.7 (63) | 27.7 (109) | 18.4 (77) | 11.1 (44) | 27.7 (128) | 20.6 (103) | 11.2 (69) |

Cognition was assessed with the Mini-Mental State Examination. Smokers and alcohol drinkers represent current consumers. z-scores and tertiles are cohort-specific. aBW, adjusted body weight; Health ABC, Health, Aging and Body Composition Study; IQR, interquartile range; LASA, Longitudinal Aging Study Amsterdam; MMSE, Mini-Mental State Examination; N85+, Newcastle 85+ Study; NuAge, Quebec Longitudinal Study on Nutrition and Successful Aging; Prot, protein; SD, standard deviation; y, years.

**Supplementary Table 5.** Association between protein intake categories (g/kg body weight/d) and walking speed (z-score) over time (β coefficients and 95%CI).

|  | **β Coefficient** | **95% CI** |
| --- | --- | --- |
| Time (years) | -0.099 | -0.106,-0.092 |
| Protein intake (g/kg BW/d) |  |  |
| <0.8 (ref.) |  |  |
| 0.8-0.99 | 0.071 | 0.022,0.108 |
| 1.0-1.19 | 0.119 | 0.047,0.159 |
| >1.2 | 0.134 | 0.074,0.172 |
| Time x Protein intake (g/kg BW/d) |  |  |
| Time x <0.8 (ref.) |  |  |
| Time x 0.8-0.99 | 0.018 | 0.008,0.029 |
| Time x 1.0-1.19 | 0.022 | 0.007,0.034 |
| Time x >1.2 | 0.025 | 0.003,0.035 |

The analytic sample consisted of 5725 participants. A joint model (hierarchical linear mixed effects and Cox proportional hazards models) was fitted to assess the association between protein intake (bodyweight not adjusted for BMI) and walking speed over time. The model is the same as model 3 in Figure. 2 and it is adjusted for categories of protein intake, sex, age and education, energy intake, smoking, alcohol intake, cognition, multimorbidity and physical activity.

**Supplementary Table 6.** Hazard ratios and 95% confidence intervals for the contribution of protein intake categories (g/kg body weight/d) to transitions in self-reported difficulty walking.

| **Protein intake** (g/kg BW/d) | | | | | | |
| --- | --- | --- | --- | --- | --- | --- |
| **<0.8** (ref.) | **0.8-0.99** | | **1.0-1.19** | | **≥1.2** | |
| HR | HR | 95% CI | HR | 95% CI | HR | 95% CI |
| **Incident mobility limitation** (n=1478) | | | | | | |
| 1.0 | 0.77 | 0.66,0.89 | 0.69 | 0.58,0.83 | 0.55 | 0.45,0.67 |
| **No mobility limitation to Death** (n=542) | | | | | | |
| 1.0 | 1.19 | 0.80,1.77 | 1.35 | 0.88,2.08 | 1.59 | 1.01,2.51 |
| **Recovery from mobility limitation** (n=742) | | | | | | |
| 1.0 | 1.08 | 0.85,1.37 | 1.45 | 1.12,1.88 | 1.44 | 1.08,1.93 |
| **Mobility limitation to Death** (n=557) | | | | | | |
| 1.0 | 1.25 | 1.04,1.51 | 1.31 | 1.06,1.62 | 1.41 | 1.12,1.76 |

Multi-state models were used to determine the association between protein intake (bodyweight not adjusted for BMI) and transitions in difficulty walking. The model is the same as model 3 in Table 2 and it is adjusted for categories of protein intake, sex, age, education, energy intake, smoking, alcohol intake, cognition, multimorbidity and physical activity.

**Supplementary Table 7.** Hazard ratios and 95% confidence intervals for the contribution of protein intake categories (g/kg body weight/d) to transitions in self-reported difficulty climbing stairs.

| **Protein intake** (g/kg BW/d) | | | | | | |
| --- | --- | --- | --- | --- | --- | --- |
| **<0.8** (ref.) | **0.8-0.99** | | **1.0-1.19** | | **≥1.2** | |
| HR | HR | 95% CI | HR | 95% CI | HR | 95% CI |
| **Incident mobility limitation** (n=1612) | | | | | | |
| 1.0 | 0.83 | 0.71,0.97 | 0.70 | 0.59,0.84 | 0.64 | 0.53,0.77 |
| **No mobility limitation to Death** (n=608) | | | | | | |
| 1.0 | 1.38 | 0.99,1.91 | 1.54 | 1.07,2.22 | 1.52 | 1.01,2.28 |
| **Recovery from mobility limitation** (n=932) | | | | | | |
| 1.0 | 1.11 | 0.90,1.37 | 1.33 | 1.05,1.69 | 1.41 | 1.09,1.82 |
| **Mobility limitation to Death** (n=598) | | | | | | |
| 1.0 | 1.09 | 0.90,1.33 | 1.14 | 0.91,1.43 | 1.27 | 1.09,1.59 |

Multi-state models were used to determine the association between protein intake (bodyweight not adjusted for BMI) and transitions in difficulty climbing stairs. The model is the same as model 3 in Table 3 and it is adjusted for categories of protein intake, sex, age, education, energy intake, smoking, alcohol intake, cognition, multimorbidity and physical activity.

**Supplementary Figure 1.** Illness-death multi-state model with the allowed transitions between no mobility limitations (walking >200m or climbing stairs), mobility limitations and death.


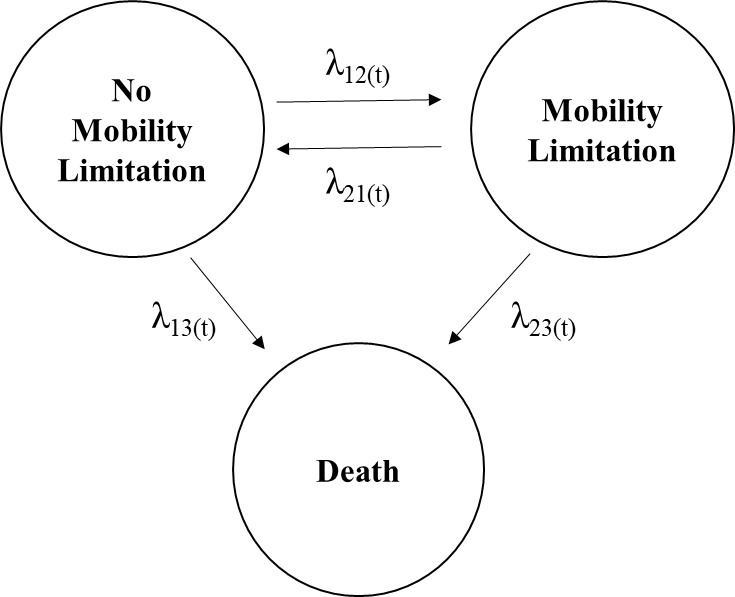


**Supplementary Figure 2.** Panel of time-series plot for walking speed, and stacked histograms for difficulty walking, climbing stairs and death over time.


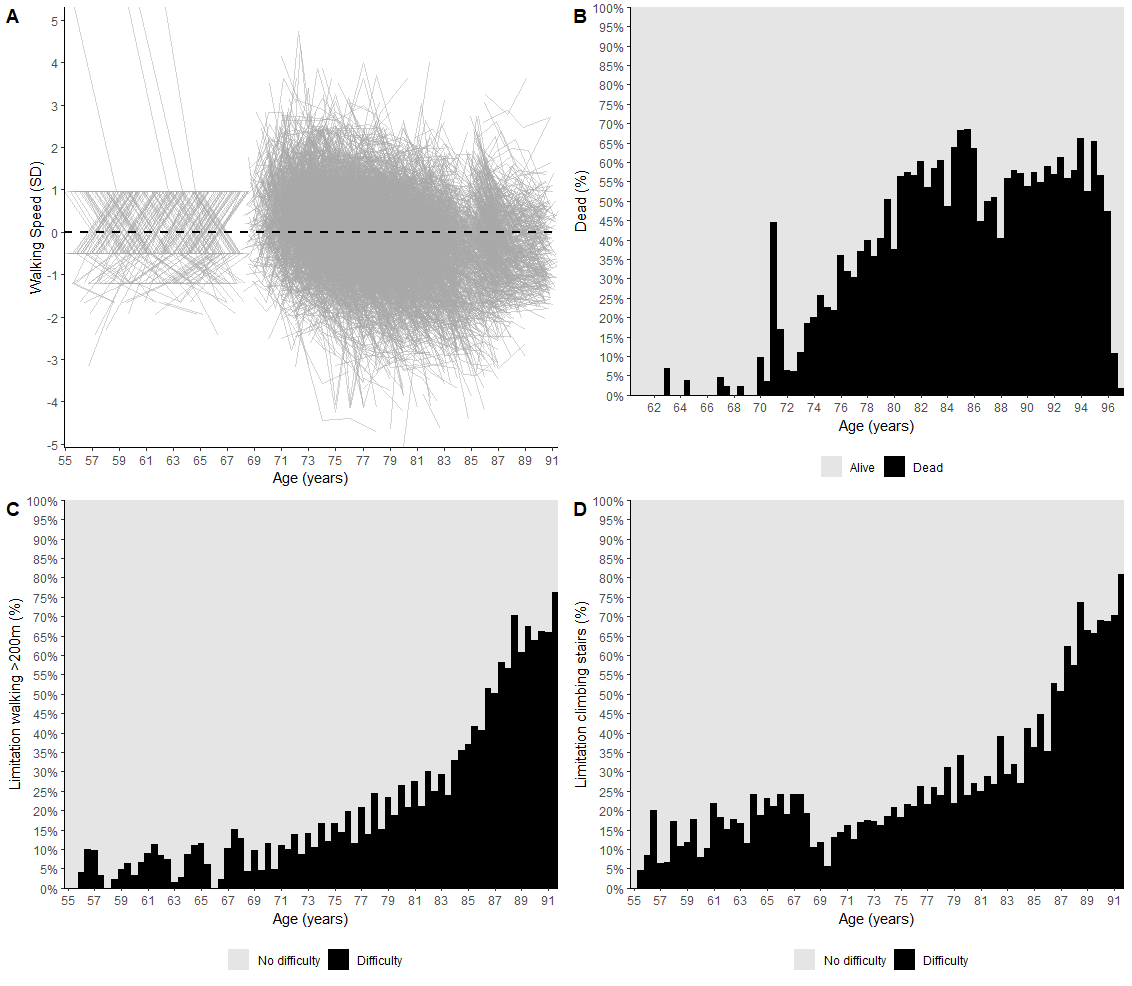


The analytic sample consisted of 5725 participants. Panel **(A)** represents the progression of walking speed z score (y axis) for every man (red) and woman (blue) over time (x axis). Panel **(B)** is the percentage of participants who reported having difficulty or being unable to walk >200 m (y axis) per age (x axis). Panel **(C)** shows the percentage of participants who reported having difficulty or being unable to climb stairs (y axis) per age (x axis). Panel **(D)** is the percentage of participants alive and dead (y axis) at a given age (x axis). The percentage of deceased participants is not cumulative.

**Supplementary Figure 3.** Association between protein intake categories (g/kg aBW/d) and walking speed (z-score) over time (β coefficients and 95%CI) by physical activity category.


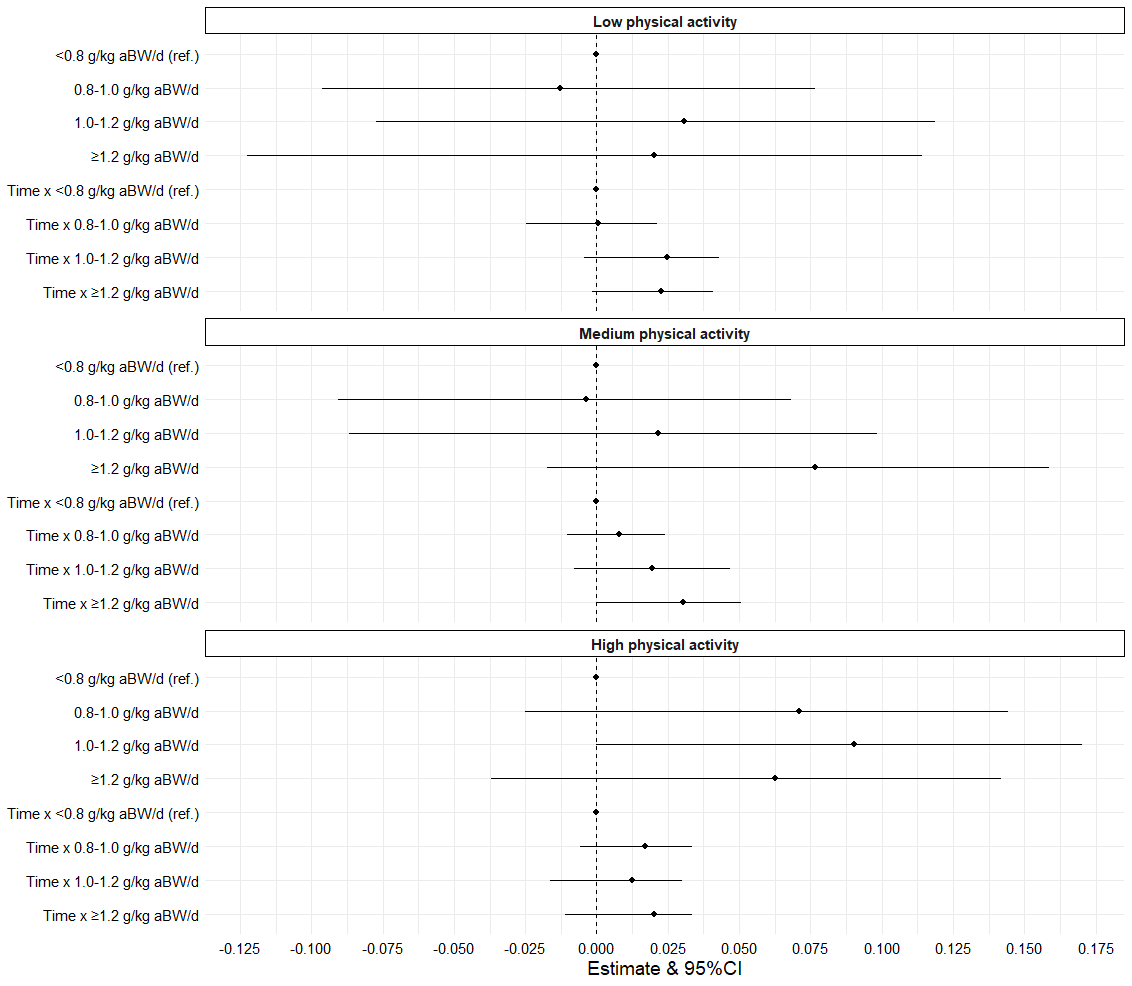
The analytic sample consisted of 5725 participants. A joint model (hierarchical linear mixed effects and Cox proportional hazards models) was fitted to assess the association between protein intake and walking speed over time stratified by physical activity. The models are adjusted for categories of adjusted protein intake, sex, age, education, energy, smoking, alcohol intake, cognition and multimorbidity and stratified by physical activity category at baseline. CI, confidence interval; educ, education; g/kg aBW/d, grams of protein per kilogram of adjusted body weight per day; ref, referent.

**Supplementary References**

1. Teng EL, Chui HC. The Modified Mini-Mental State (3MS) examination. J Clin Psychiatry 1987;48(8):314-8.

2. Folstein MF, Folstein SE, McHugh PR. "Mini-mental state". A practical method for grading the cognitive state of patients for the clinician. J Psychiatr Res 1975;12(3):189-98. doi: 10.1016/0022-3956(75)90026-6.
